# Initiation of Bladder Voiding with Epidural Stimulation in Paralyzed, Step Trained Rats

**DOI:** 10.1371/journal.pone.0108184

**Published:** 2014-09-29

**Authors:** Parag N. Gad, Roland R. Roy, Hui Zhong, Daniel C. Lu, Yury P. Gerasimenko, V. Reggie Edgerton

**Affiliations:** 1 Department of Integrative Biology and Physiology, University of California Los Angeles, Los Angeles, California, United States of America; 2 Department of Neurobiology, University of California Los Angeles, Los Angeles, California, United States of America; 3 Department of Neurosurgery, University of California Los Angeles, Los Angeles, California, United States of America; 4 Brain Research Institute, University of California Los Angeles, Los Angeles, California, United States of America; 5 Pavlov Institute of Physiology, St. Petersburg, Russia; Heidelberg University Hospital, Germany

## Abstract

The inability to control timely bladder emptying is one of the most serious challenges among the several functional deficits that occur after a complete spinal cord injury. Having demonstrated that electrodes placed epidurally on the dorsum of the spinal cord can be used in animals and humans to recover postural and locomotor function after complete paralysis, we hypothesized that a similar approach could be used to recover bladder function after paralysis. Also knowing that posture and locomotion can be initiated immediately with a specific frequency-dependent stimulation pattern and that with repeated stimulation-training sessions these functions can improve even further, we reasoned that the same two strategies could be used to regain bladder function. Recent evidence suggests that rats with severe paralysis can be rehabilitated with a multisystem neuroprosthetic training regime that counteracts the development of neurogenic bladder dysfunction. No data regarding the acute effects of locomotion on bladder function, however, were reported. In this study we show that enabling of locomotor-related spinal neuronal circuits by epidural stimulation also influences neural networks controlling bladder function and can play a vital role in recovering bladder function after complete paralysis. We have identified specific spinal cord stimulation parameters that initiate bladder emptying within seconds of the initiation of epidural stimulation. The clinical implications of these results are substantial in that this strategy could have a major impact in improving the quality of life and longevity of patients while simultaneously dramatically reducing ongoing health maintenance after a spinal cord injury.

## Introduction

The main functions of the lower urinary tract that are compromised after a spinal cord injury (SCI) are the ability to store and to expel urine in a coordinated, controlled manner [1], [2]. Two of the major problems after a SCI are an overactive bladder and/or detrusor sphincter dyssynergia (DSD) both of which lead to incomplete bladder voiding due to uncoordinated contractions of the sphincter muscles [1], [2]. Maintenance of bladder health and avoiding reflux of urine to the kidneys and other urinary tract infections are of utmost importance after a SCI. In humans, the inability to hold urine and to void when desired (incontinence) is a socially undesirable situation, although not one that is life threatening.

In the normal adult rat, storage of urine is dependent on the inhibition of parasympathetic action on the smooth bladder muscle (detrusor) and on the sympathetic tonic activation of the internal urethral sphincter for outflow resistance. During micturition, efficient voiding is dependent on synchronous activation of the detrusor muscle for contraction, relaxation of the internal urethral sphincter, and bursting activity of the striated external urethral sphincter (EUS) for enhanced urine flow [3], [4]. In humans conscious control of the initiation of these largely autonomic functions involves a complex interaction between the cerebral cortex, pontine micturition center, sympathetic and parasympathetic nervous systems, and somatic motoneurons in the lumbar spinal cord. This interaction simultaneously activates stereotypical postural adjustments that are specie as well as gender unique. Although the behavior of the EUS in rats during voiding is different from that of humans, the chronic spinal rat model demonstrates lower urinary tract dysfunction similar to that observed in other species including human subjects [6].

Given the anatomical overlap in the neural networks within the lumbosacral spinal cord that control bladder function [5], [6] and locomotor function [7], [8], [9], interactive effects of bladder function and locomotion are not surprising. For example, de Groat et al. [6] reported that the role of afferents originating in the bladder and EUS projecting to the lumbosacral region of the spinal cord was altered significantly after a mid-thoracic SCI. This overlap in the neural control of somatic and bladder function could represent either two separate neural circuits with some overlap or a single circuit being tuned differentially via varying frequencies of activation. We hypothesize that step training in the presence of electrical enabling motor control (eEmc) [10], a serotoninergic agonist (quipazine) [11] and glycinergic antagonist (strychnine) [12], [13] would reinforce the overlapping neural circuitry controlling bladder function and locomotion and that voiding could be tuned by varying the frequencies of eEmc. We further hypothesized that since bladder control is autonomic it should be more amenable to epidural modulation than the less automatic control of locomotion.

The importance of controlling voiding is that the primary danger to the paralyzed individual is bladder overfilling and not partial emptying (incontinence). The spinal rat model does not address all the issues faced by humans after a SCI due to the inability to assess voluntary vs. involuntary responses in rats. This model, however, allowed us to assess the effects of electrical enabling motor control (eEmc) on bladder voiding, since the bladder of spinal rats must be manually expressed post-injury to avoid overfilling [10], [12], [13]. The main purpose of the present study, therefore, was to assess the acute and chronic effects of varying frequencies of eEmc in the lumbosacral region of the spinal cord on bladder voiding after a complete, mid-thoracic spinal cord transection in adult female rats. In addition, the effects of step training for 6 weeks post-injury on bladder voiding were determined. The results suggest that both eEmc and step training may have positive effects on the recovery of bladder voiding in spinal rats.

## Materials and Methods

### Study design

Data were obtained from 15 (10 trained and 5 untrained) adult female Sprague Dawley rats (270–300 g body weight). Pre- and post-surgical animal care procedures have been described in detail previously [14]. The rats were housed individually with food and water provided *ad libitum*. All survival surgical procedures were conducted under aseptic conditions with the rats deeply anesthetized with isoflurane gas administered via facemask as needed. All procedures described below are in accordance with the National Institute of Health Guide for the Care and Use of Laboratory Animals and were approved by the Animal Research Committee at UCLA. All animals underwent identical surgical procedures including spinal cord transection, intramuscular EMG implantation, and spinal cord epidural electrode implantation. The rats were allowed to recover for 7 days after which step training under the influence of eEmc was initiated. Step training was performed for 6 weeks, 5 days a week for 20 min/day. At 7 weeks post-injury, the rats were tested for their ability to step bipedally on a treadmill with eEmc (40 Hz), motor evoked potentials were generated by spinal cord stimulation (1, 5, and 40 Hz) and recorded in selected hindlimb muscles and the EUS while the rat was suspended in a harness [11], [14]. Terminal bladder experiments were performed to determine the most efficient eEmc frequency to enable voiding and to study the activity of the EUS under different bladder conditions, i.e., empty, filled, and voiding. Details of each step are given below.

### Head connector and chronic intramuscular EMG electrode implantation

A small incision was made at the midline of the skull. The muscles and fascia were retracted laterally, small grooves were made in the skull with a scalpel, and the skull was dried thoroughly. Two amphenol head connectors with Teflon-coated stainless steel wires (AS632, Cooner Wire, Chatsworth CA) were securely attached to the skull with screws and dental cement as described previously [11]. Selected hindlimb muscles, i.e., the tibialis anterior (TA) and soleus, were implanted bilaterally with intramuscular EMG recording electrodes as described previously [11]. Skin and fascial incisions were made to expose the belly of each muscle. Two wires extending from the skull-mounted connector were routed subcutaneously to each muscle. The wires were inserted into the muscle belly using a 23-gauge needle and a small notch (∼0.5–1.0 mm) was removed from the insulation of each wire to expose the conductor and form the electrodes. The wires were secured in the belly of the muscle via a suture on the wire at its entrance into and exit from the muscle belly. The proper placement of the electrodes was verified during the surgery by stimulating through the head connector and post-mortem via dissection.

### Spinal cord transection, epidural electrode implantation, and post-surgical animal care procedures

A partial laminectomy was performed at the T8–T9 vertebral level to expose the spinal cord. A complete spinal cord transection to include the dura was performed at approximately the T8 spinal level using microscissors. Two surgeons verified the completeness of the transection by lifting the cut ends of the spinal cord and passing a glass probe through the lesion site. Gel foam was inserted into the gap created by the transection as a coagulant and to separate the cut ends of the spinal cord.

For epidural electrode implantation, partial laminectomies were performed to expose the spinal cord at spinal levels L2 and S1. Two Teflon-coated stainless steel wires from the head connector were passed under the spinous processes and above the dura mater of the remaining vertebrae between the partial laminectomy sites. After removing a small portion (∼1 mm notch) of the Teflon coating and exposing the conductor on the surface facing the spinal cord, the electrodes were sutured to the dura mater at the midline of the spinal cord above and below the electrode sites using 8.0 Ethilon suture (Ethicon, New Brunswick, NJ). Two common ground (indifferent EMG and stimulation grounds) wires (∼1 cm of the Teflon removed distally) were inserted subcutaneously in the mid-back region. All wires (for both EMG and epidural stimulation) were coiled in the back region to provide stress relief.

All incision areas were irrigated liberally with warm, sterile saline. All surgical sites were closed in layers using 5.0 Vicryl (Ethicon, New Brunswick, NJ) for all muscle and connective tissue layers and for the skin incisions in the hindlimbs and 5.0 Ethilon for the back skin incision. All closed incision sites were cleansed thoroughly with saline solution. Analgesia was provided by buprenex (0.5–1.0 mg/kg, s.c. 3 times/day). The analgesics were initiated before completion of the surgery and continued for a minimum of 2 days. The rats were allowed to fully recover from anesthesia in an incubator. The rats were housed individually in cages that had ample CareFresh bedding, and the bladders of the spinal rats were expressed manually 3 times daily for the first 2 weeks after surgery and 2 times daily thereafter. During bladder expressions, the urine was collected in a weigh boat and measured using a syringe to quantify the total urine manually expressed each day. The hindlimbs of the spinal rats were moved passively through a full range of motion once per day to maintain joint mobility. These procedures have been described in detail previously [11].

### Step Training

Ten rats were step trained bipedally [11] on a specially designed motor-driven rodent treadmill using a body weight support system [19] under the influence of eEmc between L2 and S1 (40 Hz) and quipazine [11] (0.3 mg/kg, i.p.) and strychnine [12], [13] (0.5 mg/kg, i.p.) at a treadmill speed of 13.5 cm/s [13]. The rats were trained for a period of 6 weeks starting one week after the spinal transection surgery. Step training in spinal rats under the influence of pharmacological and/or spinal cord stimulation interventions are routine procedures that have been performed in our lab for several years [11], [12], [13], [14].

### Testing Procedures

All 15 rats (10 trained, 5 untrained) were tested under the following conditions: 1) the rats were tested to step bipedally at a treadmill speed of 13.5 cm/s while in a body weight support system under the influence of eEmc (40 Hz bipolar stimulation between L2 and S1); 2) evoked potentials were recorded from the hindlimb muscles during bipolar epidural stimulation between L2 and S1 (1, 5, and 40 Hz) while the rats were suspended in a harness [11], [15], [16]; and 3) acute terminal experiments for bladder function were performed 7 weeks post-SCI. Under anesthesia, a PE 50 catheter was inserted via the urethra [17] and secured in place using surgical tape. The EUS muscle was implanted acutely with Teflon coated stainless steel wires (AM systems) as described previously [18]. Once the animal was conscious, they were suspended vertically using the body weight support system with their feet off the treadmill surface (Movie S2). One cc of saline was injected into the bladder via a syringe at a steady pace with no visible leakage of saline. Three trials were conducted at each stimulation frequency (1, 5, and 40 Hz). The saline voided during stimulation at each frequency of stimulation was collected in a large weigh boat positioned below the animal and then the volume measured using a syringe as described above. The bladder was expressed manually between each trial to ensure an empty bladder. No pharmacological agents were administered during the testing procedures.

### Data analysis

EMG recordings from the TA, soleus, and EUS muscles were band-pass filtered (1 Hz to 5 KHz), amplified using an A-M Systems Model 1700 differential AC amplifier (A-M Systems, Carlsborg, WA), and sampled at a frequency of 10 KHz using a custom data acquisition program written in the LabView development environment (National Instruments, Austin, TX) as described previously [11] Custom scripts written in Matlab were used to measure the evoked potentials from the hindlimb and EUS muscles [13]. Step cycle durations and EMG burst durations and amplitudes were determined using a custom program written in the LabView development environment. Burst integrated EMG (iEMG) was calculated as the area under the curve after rectification of the raw EMG signal. EMG amplitude distribution plots were generated using custom scripts written in Matlab: an L-shaped distribution reflects reciprocal activation, whereas a more D-shaped distribution reflects increased co-contraction.

### Statistical analyses

All data are reported as mean ± SEM. Statistically significant differences were determined using a one-way repeated measures analysis of variance (ANOVA) or a paired or unpaired t-test. The criterion level for the determination of a statistical difference was set at *P*<0.05 for all comparisons.

## Results

### Interaction of neural networks controlling locomotion and micturition

**Figure 1:**
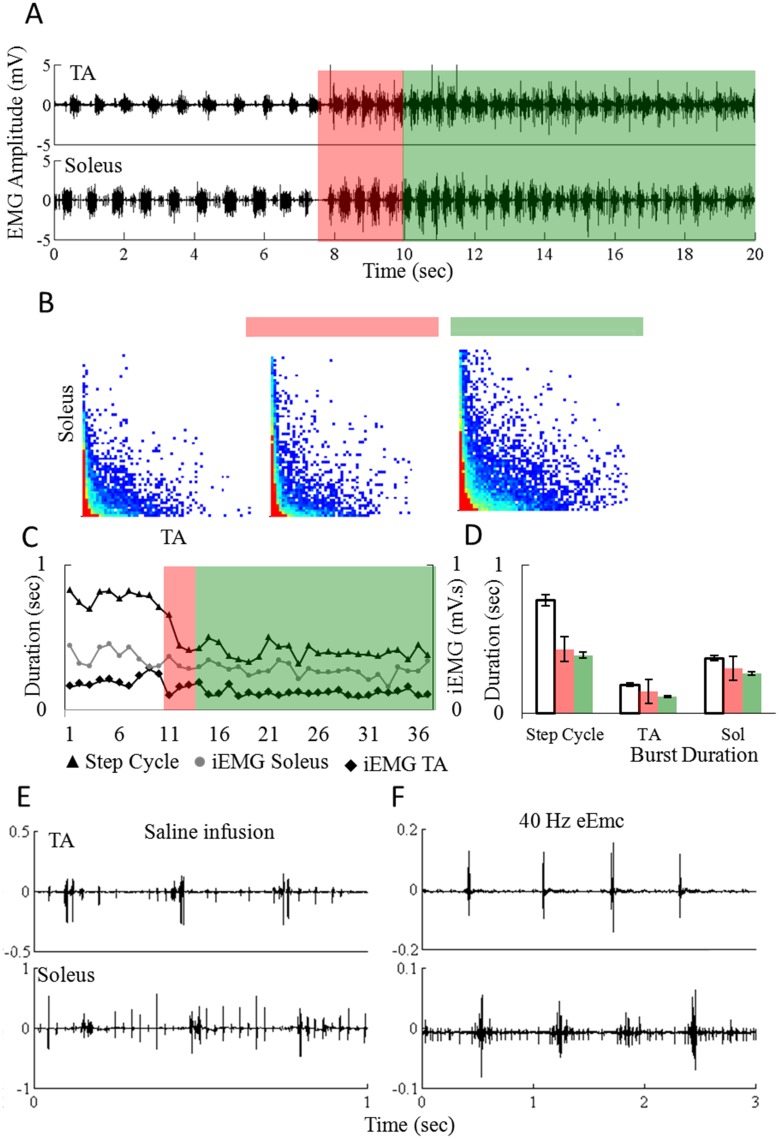
Interaction between locomotion and bladder circuits. (A) Representative EMG recordings from the soleus and tibialis anterior (TA) muscles from a spinal rat suspended in a harness and stepping bipedally on a treadmill at 13.5 cm/s under the influence of eEmc (40 Hz between L2 and S1). The rat begins to micturate at the beginning of the green shaded area. Note the shorter step cycle periods and higher EMG amplitudes a few steps prior to (red shaded area) and during (green shaded area) micturition. (B) EMG amplitude distribution plots showing an increase in the amount of co-activation of the soleus and TA muscles during the steps immediately before and during micturition. The red and green bars above the distribution plots correspond to the shaded regions in (A). (C) Step cycle duration and soleus and TA integrated EMG (iEMG) for each step shown in (A). (D) Mean (±SEM) step cycle duration and soleus and TA EMG burst durations for the regions highlighted in (A). Representative EMG recordings from the soleus and TA muscles when the rat hindlimbs were suspended above the treadmill belt (unloaded) during saline infusion (1 cc) into the bladder via a urethral catheter (E) or during 40 Hz eEmc (F).

Rats with a complete mid-thoracic spinal cord transection can step bipedally on a treadmill when the upper body is supported in a harness [19]. During this body weight supported stepping, spontaneous voiding occurs intermittently with eEmc at 40 Hz between L2 and S1 and there are distinct changes in the locomotor pattern during the transition from no voiding of urine to a state where voiding is initiated (Fig. 1A). Initially consistent stepping with reciprocal activation of the soleus and TA muscles is observed (non-highlighted region). Shortly prior to voiding (∼2 sec in this example, red highlighted region) the pattern of stepping changes (Movie S1): there is a shorter step cycle, a higher amount of co-contraction between the TA and soleus, and shorter burst durations and lower iEMG levels in both muscles compared to the prior steps (Fig. 1B–D). During voiding (green highlighted region), the step cycles become even shorter and the amount of co-contraction increases further. The corresponding EMG amplitude distribution plots (Fig. 1B) highlight the differences in the amount of co-contraction between the soleus and TA under each condition. Reciprocal activation of the soleus and TA muscles also is observed when the rat is suspended above the treadmill (no foot contact) and the hindlimbs are air stepping when saline is infused into the bladder via a urethral catheter (Fig. 1E) under the influence of 40 Hz eEmc (Fig. 1F), or when pinching the tail (data not shown). The state of the spinal locomotor circuitry, however, appears to be different under each condition. For example the EMG amplitudes in the flexors and extensors are greater and the cycle periods shorter during saline infusion compared to air stepping (40 Hz) (compare Fig. 1E and F).

### Chronic effects of step training on bladder voiding

**Figure 2:**
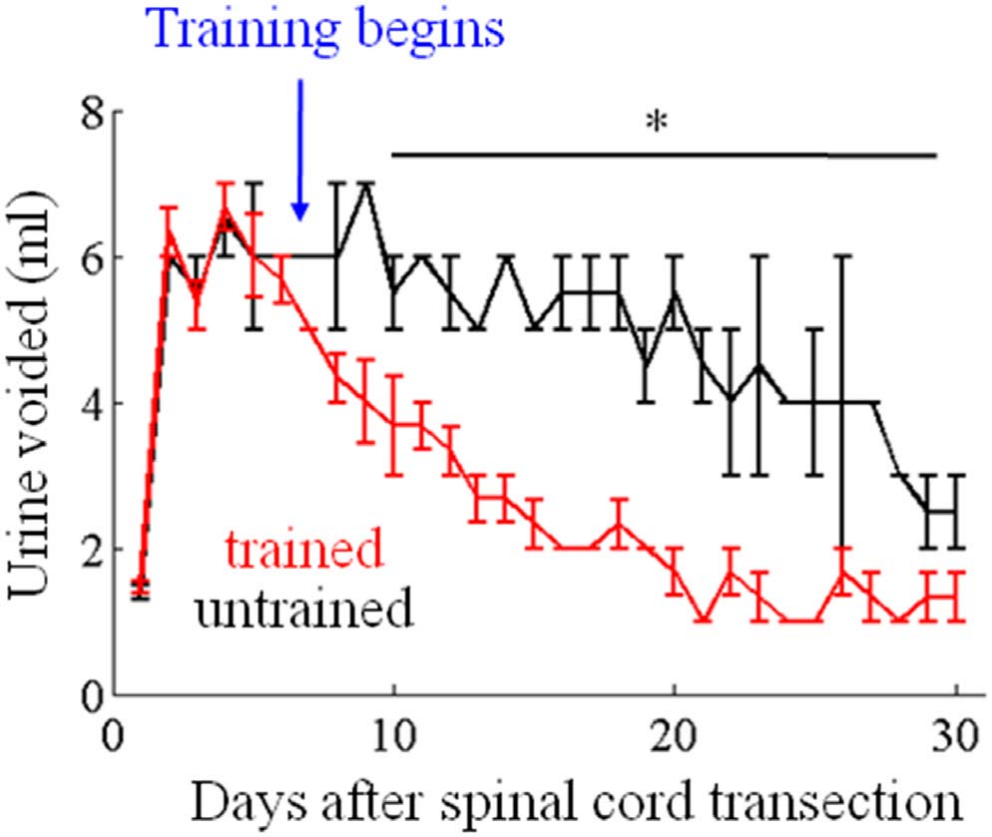
Total average volume of urine voided manually daily. Mean (±SEM) total daily volume of urine voided by manual bladder expression in spinal rats trained to step bipedally on a treadmill at 13.5 cm/s beginning 7 days post-surgery or in untrained rats (n = 3 rats/group). *, significant difference between trained and untrained at *P*<0.05.

Chronic step training under the influence of eEmc (40 Hz between L2 and S1) results in an increase in spontaneous bladder voiding both during routine cage activity as well as during treadmill stepping. The daily average volume of urine voided manually starting after 3 days of training (10 days post-SCI) and continuing for a period of 30 days was lower in step trained vs. untrained rats (Fig. 2), suggesting that the bladder was influenced by the additional sensory input to the spinal circuitry generated by load bearing itself.

### Acute effect of eEmc on bladder voiding after SCI

**Figure 3:**
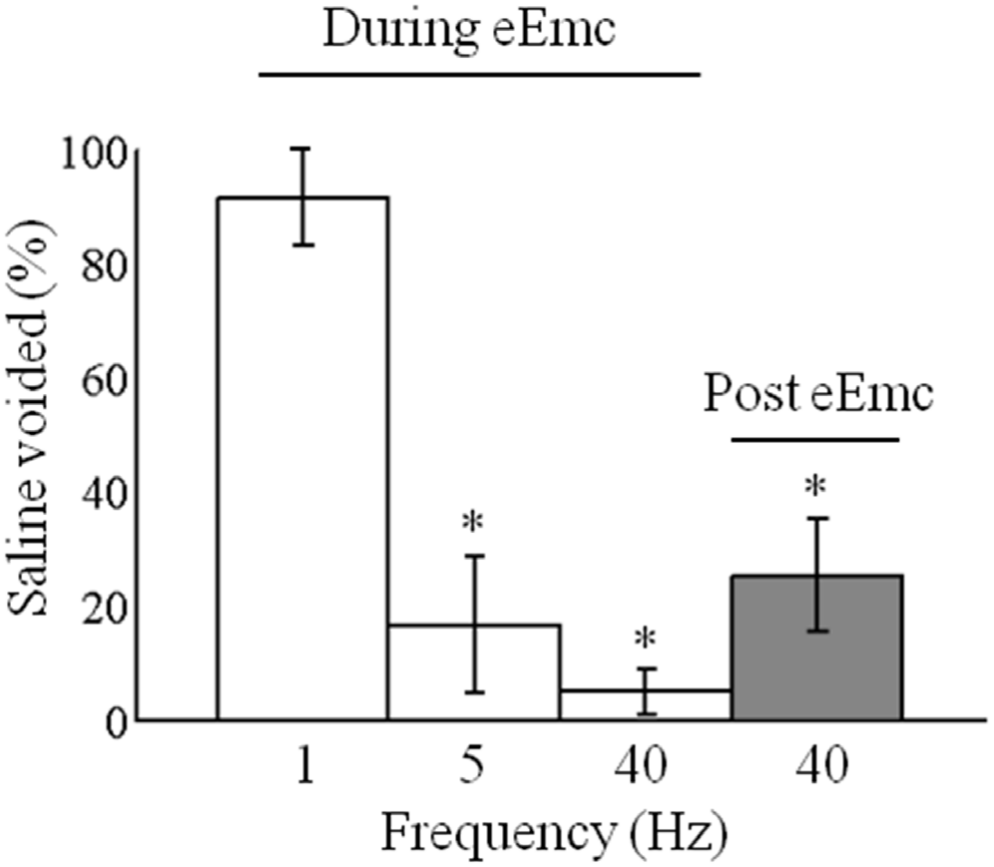
Voiding efficiency with eEmc. Mean (±SEM, n = 6 rats, 3 trials each rat) total percent volume voided in the first 90 sec after the initiation of eEmc at different frequencies after infusion of 1cc of saline via a urethral catheter in spinal rats with the hindlimbs suspended above a treadmill belt. In addition, the volume of saline voided within 30 sec after the 40-Hz stimulation was stopped (post-eEmc) is shown. *, significantly different from 1 Hz at *P*<0.05.

We determined the effects of different frequencies of stimulation on voiding by infusing controlled quantities of saline into the bladder via a urethral catheter in step trained rats that were suspended in a harness as described above. One cc of saline was infused into the bladder via the urethral catheter at a steady rate since this did not result in any leakage of saline. Any volume higher than 1cc resulted in visible leakage of saline. The most effective voiding was observed with 1-Hz stimulation (pulse width 0.5 ms, ∼2.5 V), with almost 90–95% of the volume voided within 90 sec (Fig. 3). Note that the hindlimbs show primarily a flexion motion during voiding (Movie S2). eEmc (40 Hz) resulted in very little voiding of saline (∼5%), although a rhythmic alternating bilateral locomotor pattern of the hindlimbs was observed (Fig. 1F). When stimulation at 40 Hz was stopped ∼30% of the saline was voided with no movement or evoked potentials observed in the hindlimbs. With 5-Hz stimulation strong oscillatory movements in both hindlimbs were observed but only ∼10–20% of the saline was voided.

**Figure 4:**
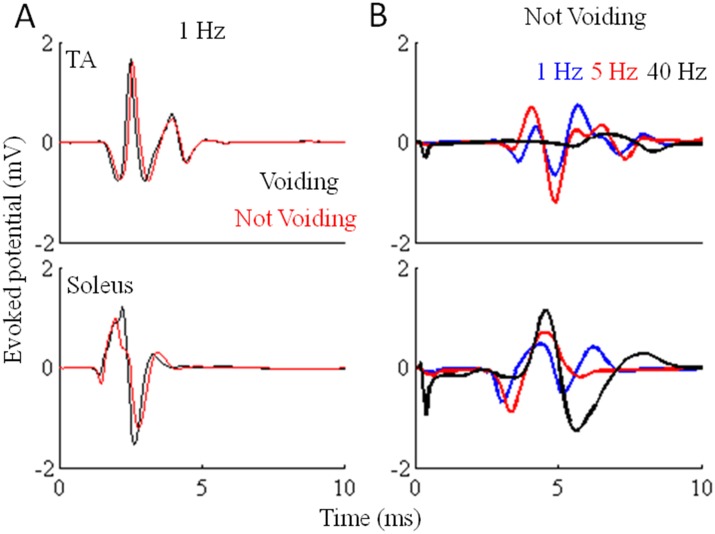
Hindlimb evoked potentials varying with eEmc and bladder voiding. (A) Mean evoked potentials (n = 4 rats, 10 potentials for each condition/rat) from the TA and soleus muscles under the influence of eEmc at 1 Hz between L2 and S1 during voiding and not voiding with a filled bladder in a spinal rat with its hindlimbs suspended above a treadmill belt. (B) Mean evoked potentials (n = 7 rats, 10 potentials for each condition/rat) in the soleus and TA induced by eEmc at 1, 5, and 40 Hz. Significant differences (*P*<0.05) in evoked potential amplitudes in the absence of voiding: TA - black < blue < red; Soleus - blue < red < black.

The responses evoked in a hindlimb extensor (soleus) and flexor (TA) muscle during 1-Hz stimulation were similar when the rats were voiding or not voiding (Fig. 4A). The mean evoked responses in the TA were higher at 1 and 5 Hz vs. 40 Hz, whereas the mean amplitude of the evoked responses was progressively higher with increased stimulation frequencies in the soleus (Fig. 4B). The observations of an increase in the flexor activation at the lower frequencies and of a high voiding efficiency at lower frequencies suggest a facilitating effect of flexion on voiding.

**Figure 5:**
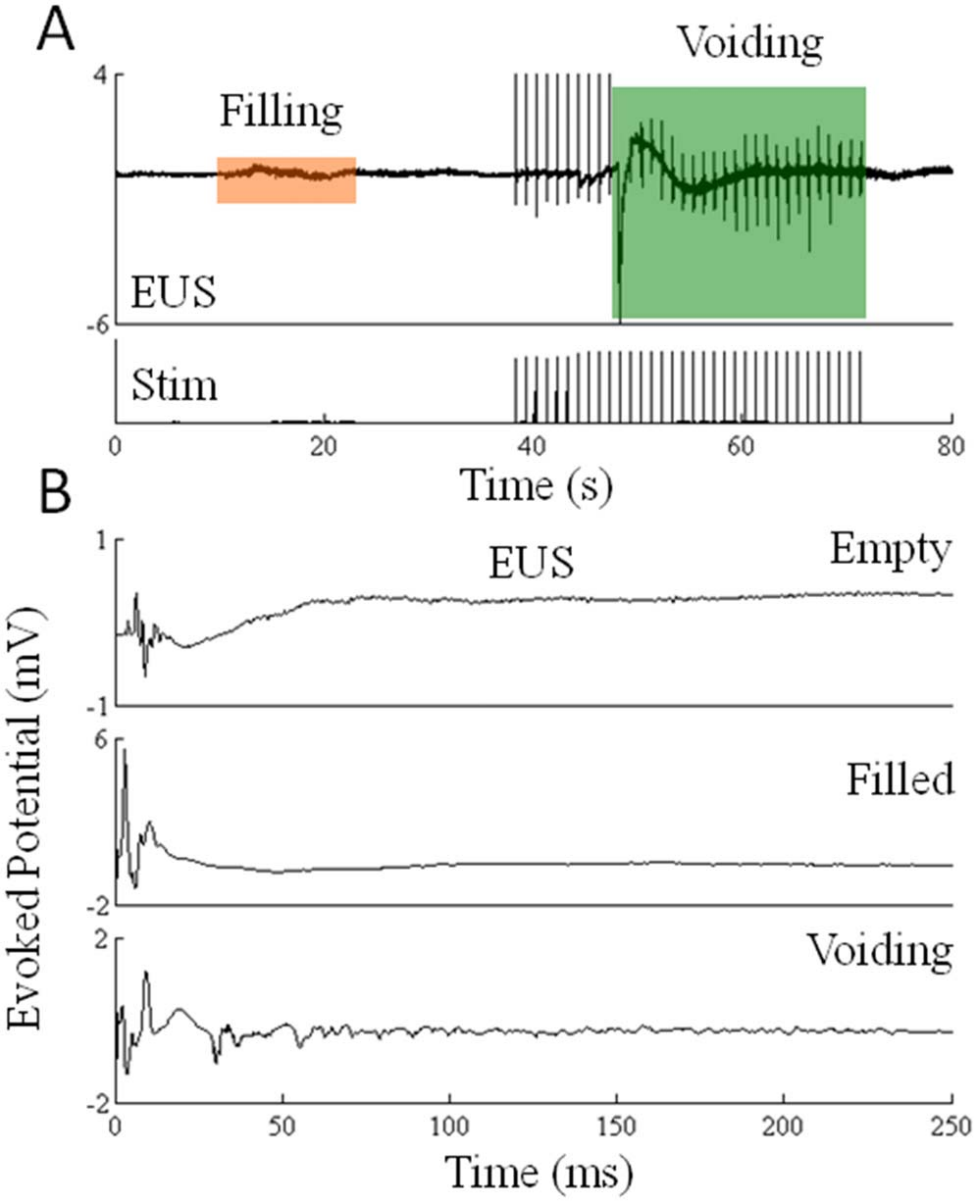
Changes in EUS activity with and without eEmc. (A) A representative EMG recording from the EUS muscle during infusion of 1 cc of saline into the bladder (orange highlight) and during voiding under the influence of 1 Hz eEmc (green highlight). (B) Average (20 potentials) evoked potentials recorded from the EUS muscle of a spinal rat at 1-Hz stimulation when the bladder was empty, filled, or voiding.

We then investigated the electrophysiological responses to 1-Hz stimulation after infusing saline into the bladder. Infusion of saline initially resulted in a slight increase in the activation of the EUS muscle followed by a period when the EUS was inactive (Fig. 5A). Stimulation at 1 Hz increased EUS activation and bladder voiding began after about 10 sec of stimulation. The evoked potentials recorded from the EUS with the bladder empty had a low amplitude with a latency of ∼3–5 ms (Fig. 5B). In comparison, these responses with a filled bladder had a higher amplitude and a shorter latency than with an empty bladder. During voiding, the EUS showed two distinct peaks with the first peak having a latency similar to that with a filled bladder but with a lower amplitude and several smaller responses with latencies varying between 20–100 ms.

**Figure 6:**
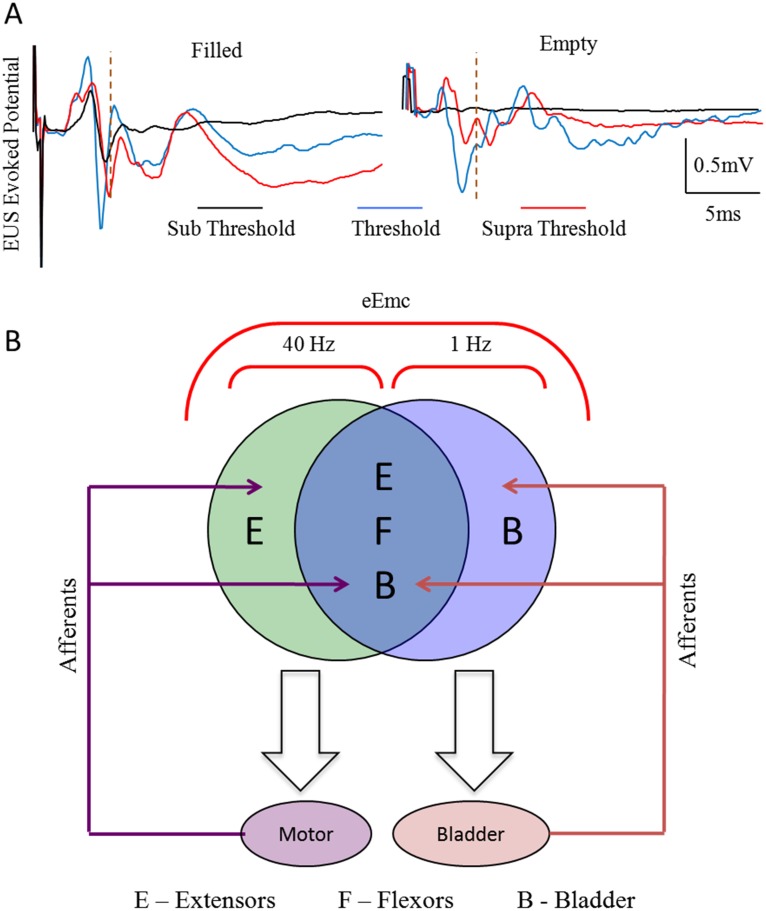
Changes in EUS evoked potential with varying intensities of eEmc. (A) Average evoked potentials from the EUS (n = 10 stimulation pulses at 1 Hz) at sub-threshold, threshold, and supra-threshold intensities of stimulation (threshold was determined based on the voltage at which voiding was initiated) with a filled bladder compared to an empty bladder. Note the presence of higher amplitude evoked potentials at a sub-threshold level with a filled bladder compared to an empty bladder and higher amplitude evoked potentials at threshold compared to supra-threshold levels with a filled bladder. Also note the higher amplitudes and longer latencies at threshold and supra-threshold levels in a filled bladder compared to an empty bladder. (B) A schematic representing the overlap of the lumbosacral spinal cord circuitries controlling locomotion and bladder voiding under the influence of eEmc and afferents from the bladder and hindlimbs is shown.

Stimulation at sub-threshold levels (threshold set at voiding intensity) with a filled bladder resulted in an evoked potential in the EUS (Fig. 6A, black trace). In addition, the amplitude and number of responses increased at voiding threshold and at supra-threshold levels of stimulation (Fig. 6A, blue and red traces, respectively). In comparison, sub-threshold stimulation (same intensity as above) with an empty bladder did not produce an evoked potential (Fig. 6A, black trace). Threshold and supra-threshold levels of stimulation, however, produced an evoked potential in the EUS, although these responses were of lower amplitude compared to those seen with a filled bladder.

## Discussion

It is well known that motor performance can be accompanied by changes of autonomic functions (somatic-visceral interactions) [20], [21]. Recent studies of human subjects with motor complete paralysis report improvement in postural control and some voluntary movement in the legs, and the subjects anecdotally have reported improved autonomic function such as bladder, sexual, and thermoregulatory control [23,23]. In this study we show that enabling of locomotor-related spinal neuronal circuits by epidural stimulation also influences neural networks controlling bladder function. Herein we have demonstrated not only the long-term complementary effects of epidural stimulation on locomotor and bladder function, but more importantly we have shown that stimulation at 1 Hz between L2 and S1 can initiate bladder emptying within seconds of the initiation of epidural stimulation. The effect of eEmc on bladder voiding on untrained rats is still not completely understood and will require further investigation.

Given the locations of the neural networks within the spinal cord that control autonomic function, the level and extent of a spinal cord lesion are important factors in the extent of loss of bladder function [24]. A complete mid-thoracic spinal cord transection in rats results in the loss of bladder function initially with some autonomically initiated spontaneous control returning in the following weeks, allowing for occasional but incomplete emptying (Fig. 2). In humans with a thoracic or cervical SCI, descending projections from supraspinal centers often are severed and their axons degenerated. In many such individuals, the reflex pathways mediating continence remain intact, but micturition cannot be initiated in a normal manner [25]. The most common form of this condition is detrusor-sphincter dyssynergia [5], [6] in which the detrusor and EUS tend to be activated together rather than reciprocally.

It appears that epidural stimulation raises the net excitability level of spinal neural networks (interneurons and motoneurons) and, when combined with motor training and/or pharmacological interventions, enhances the activation of the sensorimotor pathways that also control bladder function [22]. Data presented herein demonstrate that in addition to the chronic effects, i.e., improved spontaneous bladder emptying associated with epidural stimulation and step training, we have discovered a unique spinal epidural stimulation paradigm which can overcome detrusor-sphincter dyssynergia and induce bladder emptying on demand in unanesthesized rats with a complete mid-thoracic spinal cord transection.

### Spinal cord plasticity mediating locomotor and bladder function after a SCI

Within three days of the initiation of epidural stimulation and step training the rats started voiding spontaneously during cage activity and step training (Fig. 2). After 6 weeks of locomotor training facilitated with eEmc (40 Hz between L2 and S1) plus quipazine and strychnine administration, the spinal rats consistently voided when stimulated at 1 Hz between L2 and S1. Locomotor training [10], [12], [13] with eEmc chronically engaged the neural networks by lowering the threshold of excitability and apparently when stimulated at 1 Hz the networks were tuned to initiate micturition. An underlying mechanism of the improved spontaneous control of bladder emptying is suggested by the heightened responses of the EUS to the afferent information, i.e., indirectly sensing bladder volume (Fig. 6). More precise measurements to assess bladder function such as urodynamics and loss of fluids during exercise will be required to better understand the interaction between spinal cord stimulation, bladder voiding, and locomotor function. Similar patterns of evoked potentials have been observed in hindlimb muscles in response to eEmc [13], [16]. The enhanced spontaneous voiding of the bladder was more efficient in the trained compared to untrained rats (Fig. 2), most likely reflecting the lowered activation threshold due to the chronic engagement of the neural networks in the trained rats. This result is consistent with the observation that after 8 weeks of locomotor training facilitated by epidural stimulation and pharmacological interventions the contractile responses of the bladder to filling in anesthetized spinal rats were more efficient in trained compared to untrained animals [26]. Direct acute responses to epidural stimulation, however, were not reported.

### Frequency dependent tuning of the automaticity of the spinal cord

The complex functioning of bladder voiding was achieved via an eEmc tonic drive at 1 Hz between L2 and S1 and this was accompanied by increased flexor activity. The conceptual basis of selecting a given eEmc parameter was to activate the neural networks that initiate the automaticity of the coordinated contractions and relaxations of the bladder and EUS that result in bladder voiding. During stimulation at 1 Hz, the bladder contracts tonically. This response occurs in the EUS within a 20 to 100 ms time window (Fig. 5B). These evoked potentials with a long latency may represent the activation of complex interneuronal networks that enable bursting activity of the EUS similar to that observed during voiding in intact rats [18]. Stimulation at higher frequencies (40 Hz) at the same sites on the spinal cord (between L2 and S1) facilitates partial weight-bearing bipedal [10] and quadrupedal [27] stepping of spinal rats on a treadmill belt. A consistent bilateral air stepping pattern also is observed when the rats are suspended in a harness and under the influence of eEmc at 40 Hz and with sufficient levels of current [13] (Fig. 1F). Similar bilateral air stepping-like patterns were observed in the hindlimbs during infusion of saline and tail pinching, suggesting that the afferent information from the bladder and EUS being processed by the neural networks in the lumbosacral region of the spinal cord contribute to these hindlimb responses. These results provide further evidence of an overlap between the networks controlling bladder function and locomotion.

### Neural networks controlling locomotion and micturition

Over the past several decades, multiple techniques have been used to induce micturition after SCI, including stimulation of the bladder wall, the pelvic nerve, and/or the sacral nerve. Directly stimulating the bladder wall induces local contractions, but high currents or a large number of electrodes are needed to induce a more widespread contraction to achieve sufficient bladder emptying. Pelvic nerve stimulation has been shown to contract the bladder wall, but as the pelvic nerve does not innervate the EUS, minimal effect was seen on the EUS resulting in a low voiding efficiency [28]. Voiding was achieved, however, by cutting the pudental nerve. This largely irreversible procedure eliminates sensation from the external genitalia of both sexes and the skin around the anus and perineum, as well as the motor supply to various pelvic muscles, including the EUS and the external anal sphincter. Sacral nerve stimulation seemed to offer the best results, but this requires complicated surgical procedures and a serious risk of permanent damage due to the intradural approach [29]. Recently a closed-loop neuroprosthesis interface was used to measure bladder fullness through implanted afferent dorsal rootlets into microchannel electrodes to measure and interpret sensory activity related to bladder fullness in spinal rats [30]. Voiding was achieved using low frequency anterior sacral nerve stimulation. While promising, the viability of this chronically implanted dorsal root-microchannel electrode system in humans has yet to be established.

Each of the above strategies involves surgically severing nerves, result in permanent loss of some motor and sensory functions, and directly induce contractions in the bladder. Therefore, these strategies completely bypass the automaticity that is intrinsic to the sensorimotor neural circuitries present in the spinal cord. While epidural electrode array implantation also is invasive, similar surgical procedures are followed in routinely performed procedures to alleviate chronic pain. Moreover, no indwelling electrodes in spinal nerves or irreversible surgical denervation of nerves or rootlets are necessary. By preserving the sensorimotor networks that underlie the automaticity of micturition, as occurs with the recovery of stepping and standing after a complete spinal cord transection, bladder function can be largely re-established by enabling the inherent automaticity present within the spinal cord [9], [31]. The spinal cord circuitry contains the necessary circuitry to control bladder voiding when provided the appropriate afferent information from the bladder and the EUS. Based on frequency-specific stimulation parameters, the spinal cord can be tuned to enable the appropriate physiological responses (Fig. 6). The clinical implications of this technique are immense and parallel the impact that spinal cord stimulation has had on the recovery of locomotion and postural abilities and some volitional movement after complete paralysis in humans [22], [23]. The application of spinal cord stimulation to overcome difficulty in micturition is not likely limited to SCI but could be adapted to other neurological disorders. This protocol could be invaluable in avoiding unnecessary transurethral catheterization, potentially lowering the incidence of urinary tract infections, and reversing the uptake of urine into the kidney.

In summary, the main findings include 1) the demonstration of functional links between the neural control (biomechanical and electrophysiological) of locomotion and micturition in awake unanesthetized rats, 2) the immediate effect of *in vivo* spinal cord stimulation on micturition, and 3) the positive chronic effects of step training under the influence of eEmc and pharmacological interventions on bladder function.

## Supporting Information

Movie S1 **A representative rat with a complete mid-thoracic spinal cord transection stepping on a treadmill at 13.5 cm/s under the influence of eEmc (40 Hz between L2 and S1) spontaneously empties its bladder.** The bladder voiding is accompanied by a change in pattern of stepping and corresponding EMG. Note the pattern change starts ∼2 sec prior to the initiation of voiding.
(MP4)

Movie S2 **A representative rat with a complete mid-thoracic spinal cord transection suspended in a harness voids its bladder under the influence of eEmc at 1 Hz between L2 and S1.**
(MP4)
